# Co-Formulation of Pembrolizumab Murine Surrogate RMP1-14 with Imagent^®^ Ultrasound Contrast Agent Enhances Intratumoral Antibody Delivery Through a Transient Increase in Tumor Blood Perfusion

**DOI:** 10.3390/pharmaceutics18060690

**Published:** 2026-05-31

**Authors:** Imani A. Kirven, Patrice Penfornis, Muhammad R. Siddiqui, Kenneth R. Butler, Richard J. Roman, Clayton T. Larsen, Candace M. Howard, Pier Paolo Claudio

**Affiliations:** 1 Department of Pharmacology & Toxicology, University of Mississippi Medical Center, Jackson, MS 39216, USA; ikirven@umc.edu (I.A.K.); ppenfornis@umc.edu (P.P.); kbutler@umc.edu (K.R.B.); rroman@umc.edu (R.J.R.); 2 University of Mississippi Medical Center, Jackson, MS 39216, USA; msiddiqui1@umc.edu; 3 Vesselon, Inc., Wilton, CT 06897, USA; clay.larsen@vesselon.com; 4 Department of Radiology, University of Mississippi Medical Center, Jackson, MS 39216, USA; cmhoward@umc.edu

**Keywords:** ultrasound contrast agents, microspheres, microbubbles, liposomes, ultrasound, checkpoint inhibitors, colorectal cancer, therapeutic index

## Abstract

**Background/Objectives**: Immune checkpoint inhibitors targeting the PD-1/PD-L1 axis have transformed cancer treatment, yet therapeutic responses remain limited in many solid tumors due to poor and uneven drug distribution within the tumor microenvironment (TME). Here, we evaluated whether co-formulation of an anti-PD-1 antibody (RMP1-14, murine surrogate for pembrolizumab) with Imagent^®^ microbubble/liposome (MBLP) complexes and ultrasound activation could enhance tumor-specific delivery while reducing systemic exposure. **Methods**: Immunocompetent MC-38 colorectal tumor-bearing mice (B6(Cg)-Tyrc-2J/J, 7-week-old females) received isotype control, isotype/MBLP/US, RMP1-14 alone, RMP1-14/MBLP, or RMP1-14/MBLP/US. Survival was analyzed by Kaplan–Meier curves, tumor necrosis by H&E staining, antibody biodistribution by immunohistochemistry, and tumor perfusion by laser speckle imaging. **Results**: No significant differences in tumor size or body weight were observed between groups. Survival analysis showed significant improvements in the RMP1-14 (*p* = 0.013) and RMP1-14/MBLP/US (*p* = 0.047) groups versus isotype controls, with the RMP1-14/MBLP/US group achieving the longest mean survival (57.8 days vs. 26.5 days for RMP1-14 alone) and complete tumor regression in 2/8 mice. The RMP1-14/MBLP/US group demonstrated significantly greater tumor necrosis than all other groups. Immunohistochemical analysis confirmed a 6.1-fold increase in intratumoral antibody accumulation with MBLP/US versus RMP1-14 alone (*p* = 0.0003), alongside significantly reduced off-target exposure in spleen, liver, kidney, and heart. Laser speckle imaging revealed a transient ~30% increase in tumor perfusion during MBLP/US treatment, consistent with cavitation-mediated hemodynamic effects. **Conclusions**: These findings demonstrate that MBLP/US co-formulation enhances intratumoral delivery of checkpoint inhibitors, improves survival, and reduces systemic organ exposure, representing a promising platform to improve the efficacy and safety profile of antibody-based immunotherapy.

## 1. Introduction

Colorectal cancer (CRC) is the third most common malignancy and the second leading cause of cancer-related mortality worldwide, accounting for approximately 1.9 million new cases and 900,000 deaths annually [1,2]. Despite advances in cytotoxic chemotherapy, radiotherapy, surgical resection, and molecularly targeted agents, the five-year survival rate for metastatic CRC remains around 15%, with durable responses only achieved in a minority of patients [2,3,4]. These discouraging statistics underscore the urgent need for therapeutic strategies to overcome the intrinsic resistance mechanisms driving CRC progression.

The emergence of immune checkpoint inhibitors (ICIs) targeting the programmed cell death-1 (PD-1)/PD-L1 axis has transformed oncology, producing long-lasting responses across multiple tumor types, including subsets of patients with several tumor types such as CRC, melanoma, non-small cell lung cancer (NSCLC), and renal cell carcinoma [5,6,7]. Pembrolizumab, an antibody against human PD-1, and its preclinical murine surrogate RMP1-14, function by blocking inhibitory signaling on cytotoxic T lymphocytes, thereby restoring their capacity to recognize and eliminate tumor cells [8,9]. However, despite regulatory approval for mismatch repair-deficient (dMMR) CRC, 90% of CRC tumors are microsatellite stable (MSS) and exhibit primary resistance to PD-1 blockade, with objective response rates below 5% in unselected populations [10,11,12]. Even in responsive patients, immune-related adverse events driven by off-target antibody distribution in lymphoid organs and healthy tissues can be dose-limiting and necessitate discontinuation of immunotherapy [13,14,15]. These dual challenges in insufficient intratumoral efficacy and excessive systemic exposure define the central therapeutic dilemma facing checkpoint-based immunotherapy in CRC.

A principal barrier to effective intratumoral ICI delivery via intravenous administration is the physical architecture of solid tumors. Elevated interstitial fluid pressure, aberrant tumor vasculature, a dense extracellular matrix, and heterogeneous blood perfusion collectively impede the extravasation and deep stromal penetration of larger macromolecules such as monoclonal antibodies [16,17,18]. The resulting concentration gradients favor perivascular antibody accumulation while leaving immunologically critical regions of the tumor microenvironment (TME) effectively undertreated [16,19]. Antibodies that do not effectively penetrate the tumor microenvironment remain in the circulation and redistribute to highly perfused organs, including lymphoid tissues, liver, spleen, and kidneys, as shown in biodistribution studies of anti-PD-1/PD-L1 antibodies [20,21,22,23]. Accumulation and immune activation in these non-tumor tissues are thought to contribute to systemic immune-related toxicities such as hepatitis and nephritis observed with checkpoint inhibitor therapy [20,24,25]. Strategies capable of simultaneously improving intratumoral antibody distribution and reducing off-target organ exposure would therefore represent a meaningful advance for this class of therapeutics.

Ultrasound-targeted microbubble delivery (UTMD) has emerged as a non-invasive, image-guided, clinically translatable strategy to overcome the barriers observed in ICI [26,27,28]. MBs are gas-filled, phospholipid-shelled particles ranging from 1–10 μm in diameter that are acoustically active and can be co-administered with drugs or chemically formulated with a wide range of therapeutic payloads [29]. Upon intravenous administration, MBs circulate through the vasculature and, upon exposure to acoustic energy at the target tissue, undergo cavitation. This cavitation is a process that encompasses stable oscillation at lower mechanical indices and which, at higher ultrasound mechanical indices, causes inertial collapse of the MBs [30]. The resulting microstreaming, microjets, and transient pore formation in vascular endothelium through sonoporation collectively increase local vascular permeability, reduce interstitial pressure, and enhance extravascular and interstitial transport of co-administered micro- and macro-molecules [26,31,32]. Most importantly, these biophysical effects are spatially confined to the sonicated region, thereby limiting collateral effects to surrounding healthy tissue [30,31]. Beyond delivery enhancement, acoustic cavitation has been shown to stimulate immunogenic cell stress, promote dendritic cell maturation, and augment CD8+ T lymphocyte infiltration into the TME, effects that could act synergistically with PD-1 blockade to amplify antitumor immune responses [27,33,34].

Our laboratory has extensively characterized the Imagent^®^ MBLP platform, an FDA-approved lipid-encapsulated, perfluorohexane-filled microbubble, in the presence of excess lipids, as a versatile co-formulation vehicle for targeted delivery of viral gene therapy [35,36,37]. We have demonstrated that Imagent^®^ MBs can protect oncolytic viral payloads (OVs) from detection and rapid degradation by the host immune system during intravenous administration, enabling systemic delivery without inducing neutralizing antibodies or activating the innate immune system [38]. We further showed that MB-mediated ultrasound delivery achieves a more homogenous intratumoral distribution than direct intratumoral injections, and that this platform can be applied across multiple payload classes [35,39]. In addition, we reported that MB encapsulated OV complexes targeted by sonoporation produced significantly greater tumor necrosis and CD8+ T lymphocyte infiltration with reduced regulatory T-cell presence in humanized mice bearing triple-negative breast cancer xenografts, including in the context of adjuvant pembrolizumab treatment [39]. These findings collectively establish a mechanistic and translational foundation for extending this biophysical MB-US platform to the targeted delivery of ICIs in immunocompetent tumor models utilizing both the microbubbles and liposomes that are self-assembled and encapsulate ICIs when reconstituting Imagent^®^ perfluorohexane lipid microsphere powder in the presence of the drug diluent.

Despite growing interest in MB-mediated ultrasound immunotherapy delivery, a critical gap remains: few studies have simultaneously and quantitatively assessed antibody bioavailability across the TME, off-target organ exposure, tumor hemodynamics, and long-term survival outcomes in a single immunocompetent in vivo model. Without this integrated evidence, it is difficult to determine whether observed therapeutic benefits arise from enhanced local delivery of the ICI or biophysical modulation of the TME, or from the combination of both. In this study, we directly address this question using an immunocompetent syngeneic MC-38 colorectal adenocarcinoma model in C57BL/6 background mice. We used the RMP1-14 anti-mouse PD-1 antibody as a diluent for reconstituting Imagent^®^ and evaluated the therapeutic effects of MBLP-mediated ultrasound delivery on mouse survival, tumor necrosis, antibody biodistribution in tumors and major organs by immunohistochemistry, and tumor blood perfusion by laser speckle imaging. In all, this provides a novel, comprehensive, mechanistically linked characterization of this delivery approach in the CRC model within an intact immune system.

## 2. Materials and Methods

### 2.1. Cell Culture

MC-38 murine colorectal cancer cells obtained from the American Type Culture Collection (ATCC, Rockville, MD, USA) were labeled with firefly luciferase through lentiviral transduction and selected by puromycin and flow cytometry to exclude inefficiently transduced cells for in vivo experiments. MC-38-LUC-sel cells were grown in Dulbecco’s modified Eagle’s medium (Innovative Research, Novi, MI, USA) supplemented with 10% FBS (Avantor, Radnor, PA, USA), 100 units/mL penicillin (Gibco, Grand Island, NY, USA), and 1 mg/mL streptomycin (Gibco, Grand Island, NY, USA). Cells were maintained at 37 °C, in a 5% CO_2_ incubator.

### 2.2. Antibodies

The in vivo PLATINUM™ functional-grade preparations of anti-mouse PD-1 monoclonal antibody (clone RMP1-14, Cat# P372) and the isotype control (Rat IgG2a, Cat# R1367) with an initial concentration of ≥5 mg/mL, were obtained from Leinco Technologies (St. Louis, MO, USA) and were stored at 4 °C prior to use.

### 2.3. Microbubble Formulation

Imagent^®^ perfluorohexane lipid microspheres (Vesselon, Wilton, CT, USA) are FDA-approved ultrasound contrast agents composed of a phospholipid shell encapsulating a perfluorohexane and nitrogen mixture. Microbubbles are stored at room temperature as a spray-dried powder under a gas headspace that is reconstituted with sterile water for injection. Each vial of reconstituted Imagent^®^ suspension contains an average of 1.37 × 10^10^ microbubbles with a volume-weighted median diameter of 6 μm. In addition, excess lipids allow immediate self-assembly of 50 trillion liposomes at room temperature in less than 5 min. This encapsulation process is performed on-site and does not require any pre-loading of drugs at the site of manufacture. Imagent^®^ microbubble/liposomes were reconstituted at room temperature with 5 mL of 1 mg/mL RMP1-14 or Rat IgG2a, yielding a formulation of three components comprising free antibody, liposome-encapsulated antibody, and antibody-linked microbubbles.

### 2.4. Mouse Model

Animal studies were conducted in accordance with the National Institutes of Health guidelines and following approval by the Institutional Animal Care and Use Committee (IACUC) at the University of Mississippi Medical Center (IACUC protocols #2023-1275 and #2026-1462). Humane animal care and mouse treatment were in strict compliance with (i) institutional guidelines, (ii) the Guide for the Care and Use of Laboratory Animals (National Academy of Sciences, Washington, DC, USA, 1996), and (iii) the Association for Assessment and Accreditation of Laboratory Animal Care International (Rockville, MD, USA, 1997).

For our studies, we utilized 7-week-old female immunocompetent B6(Cg)-Tyrc-2J/J (Strain#000058, The Jackson Laboratory, Bar Harbor, ME, USA), which are C57BL/6J background mice carrying a mutation in the tyrosinase gene, resulting in the complete absence of pigment in skin, hair, and eyes. Mice were acclimated for a week following their arrival at the animal facility before starting the study.

Survival Study: Forty-five B6(Cg)-Tyrc-2J/J mice were subcutaneously allografted with 1 × 10^5^ MC-38-LUC-sel CRC cells suspended in PBS and Matrigel (Corning Incorporated, Corning, NY, USA) at a 1:1 ratio in their right flanks. Tumor size on all mice was monitored on the same day weekly using a caliper and an AMI HT/HTX bioluminescence optical imager (Spectral Instruments Imaging, Tucson, AZ, USA).

Upon tumors reaching 150–200 mm^3^ by caliper measurement, mice were randomized by body weight and assigned to one of the following 5 groups: (A) Isotype; (B) Isotype/MBLP/US; (C) RMP1-14/MBLP/US; (D) RMP1-14; and (E) RMP1-14/MBLP. Once assigned to the groups, mouse ears were punctured for identification. To minimize potential confounding factors and reduce experimental bias, all procedures were standardized whenever possible, including having the same trained investigator perform procedures and outcome assessments under identical protocols and environmental conditions. Animals were randomly allocated by weight to experimental groups, and cage position, treatment administration order, and measurement order were counterbalanced when feasible to ensure that all groups were similarly affected by potential sources of variability. The same trained investigator who performed all procedures was aware of the grouping of the mice during treatment. However, all outcome assessments and data analysis were blinded.

Mice were anesthetized using a VetEquip IMPAC6 anesthesia machine (VetEquip Inc., Marsing, ID, USA) and transferred to a BSL2 hood on a heating pad. A continuous flow of isoflurane was provided through a nose cone during the procedure. Mice were treated according to the randomized groups as follows: mice in all five groups received three intravenous injections, spaced over the course of 8 days of either isotype control, isotype/MBLP/US, RMP1-14/MBLP, RMP1-14, or RMP1-14/MBLP/US using an insulin syringe with a 30G needle. The mice received 100 μL of RMP1-14 or isotype (at a dose of 100 μg/mouse) in the lateral tail vein. Groups B and C injections were followed by US directly on the tumor for 7 min using a Mindray TE7 ultrasound machine (Mindray, Mahwah, NJ, USA) with a 2.2 MHz probe, set to 0.33 Mechanical Index (MI), with a transient pulse to 1.3 MI every 10 s. The experimental endpoint was determined by a tumor size restraint of 2000 mm^3^ or the onset of a tumor ulceration. Upon reaching either endpoint, mice were sacrificed, and tumors, livers, spleens, kidneys, hearts, lungs, and blood were collected and fixed in 10% neutral buffered formalin for further pathological analysis.

### 2.5. Antibody Distribution Study

In each group, eight B6(Cg)-Tyrc-2J/J mice received either a single IV injection with saline, 100 μg/mouse/100 μL RMP1-14 alone, or RMP1-14 with MBLP/US. Three hours post-treatment, mice were sacrificed, and tumors, spleens, livers, kidneys, and hearts were harvested and fixed in 10% neutral buffered formalin for further immunohistochemical analysis.

### 2.6. Hematoxylin and Eosin Staining and Immunohistochemical Analysis

Tumors were dissected, processed for paraffin embedding, and sectioned into 7 µm sections by the University of Mississippi Medical Center Histology Core. Hematoxylin and Eosin (H&E) staining was performed for histopathological evaluation, including assessment of necrotic regions. Tumor size and necrotic regions were independently measured by three blinded observers using consistent morphometric criteria. Immunohistochemical staining using avidin-biotin-peroxidase methodology was performed in accordance with the manufacturer’s instructions (Vectastain ABC Elite Kit, Vector Laboratories, Burlingame, CA, USA). Our modified protocol includes deparaffination in xylenes, rehydration through descending grades (100%, 95%, 90%, 85%, and 70%) of alcohol, up to water, followed by non-enzymatic antigen retrieval with 0.01 M sodium citrate buffer (Millipore Sigma, Burlington, MA, USA) pH 6.0 at 95 °C for 18 min under high pressure, endogenous peroxidase quenching with 3% H_2_O_2_ for 10 min at room temperature, and blocking with 10% normal goat serum. To selectively detect intravenously injected RMP1-14, with or without MBLP-mediated US delivery, and to assess its tissue distribution after 3 h, slides were incubated overnight at room temperature in a humidified chamber with biotinylated goat anti-rat IgG2a (R1215, Lienco Technologies, St. Louis, MO, USA) at a 1:500 dilution. After rinsing in phosphate-buffered saline (PBS), sections were incubated with the avidin–biotin–peroxidase complexes for 45 min at room temperature. Finally, the peroxidase was developed with diaminobenzidine (Vector Laboratories, Burlingame, CA, USA) for 8 min, and the sections were counterstained with hematoxylin and mounted with Permount (Fisher Scientific, Pittsburgh, PA, USA). Slides were scanned using a Philips Ultra-Fast Scanner 1.6 RUO and the Philips Image Management System (IMS) prior to threshold analysis. Positive antibody area was quantified using ImageJ 1.54p color deconvolution with the H-DAB setting, which separates the hematoxylin and DAB channels, enabling isolated thresholding of the DAB-positive signal. A standardized threshold was applied uniformly across slides, and inter-observer reliability was confirmed by three blinded observers.

### 2.7. Tumor Blood Perfusion Assessment

In each group, eight B6(Cg)-Tyrc-2J/J mice were anesthetized using intramuscular 50 mg/kg ketamine (Dechra Veterinary Products, Overland Park, KS, USA) and intraperitoneal 150 mg/kg Inactin hydrate (Sigma-Aldrich, St. Louis, MO, USA). A cruciform incision was used to expose the tumor surface to visualize tumor blood perfusion using the Laser Speckle Imager (RFLSI III, RWD Life Science, Shenzhen, China). Regions of interest (ROIs) were placed at various locations on the tumor to assess perfusion at the tumor surface, and continuous images were captured at 1-s intervals. After baseline measurements were taken, mice received either a single IV injection of 100 μL of saline or of the Imagent^®^ contrast agent. Injections were followed by sonoporation with a 2.2-MHz probe at 0.33 MI. Perfusion was measured with and without a transient pulse of 1.3 MI every 10 s. At the end of the experiment, mice were sacrificed, and tumors were harvested and fixed in 10% neutral buffered formalin for further pathological analysis.

### 2.8. Statistical Analysis

Power analysis for two-way ANOVA indicated that 8 mice/group would provide 95% power (α = 0.05) to detect a ≥20% difference in intratumoral antibody accumulation between ultrasound-targeted and untargeted control groups to determine the number of B6(Cg)-Tyrc-2J/J required for our study. This difference has been observed in similar comparisons by experiments performed in our laboratory [39]. Using GraphPad Prism 10 statistical software (GraphPad, Inc., La Jolla, CA, USA), statistical analysis performed one- and two-way analysis of variance (ANOVA) with Tukey or Bonferroni post hoc tests, and two-tailed *t*-tests were used to determine the statistical significance of differences between experimental groups. *p*-values of less than 0.05 were considered statistically significant.

## 3. Results

### 3.1. MBLP/US Improves RMP1-14-Mediated Survival Outcomes

**Figure 1:**
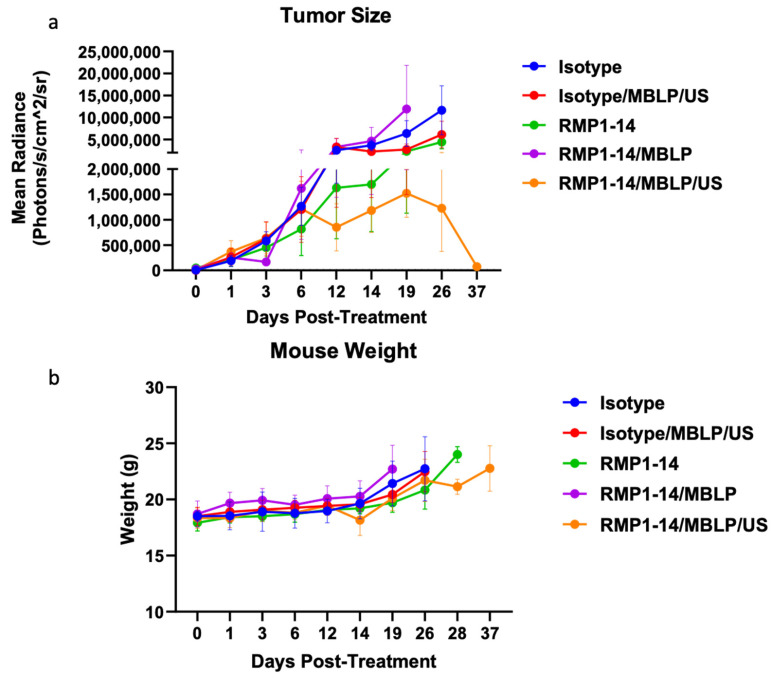
Tumor growth and body weight over time following treatment. (**a**) Longitudinal tumor burden measured by bioluminescence imaging (mean radiance, photons/s/cm^2^/sr) across treatment groups over 37 days following treatment. (**b**) Corresponding mouse body weights (g) were monitored across the same time course to assess treatment tolerability. Groups (number of mice/group = 8) include Isotype control, Isotype/MBLP/US, RMP1-14, RMP1-14/MBLP, and RMP1-14/MBLP/US. Data are presented as mean ± SEM. MBLP, microbubbles liposomes; US, ultrasound.

**Figure 2:**
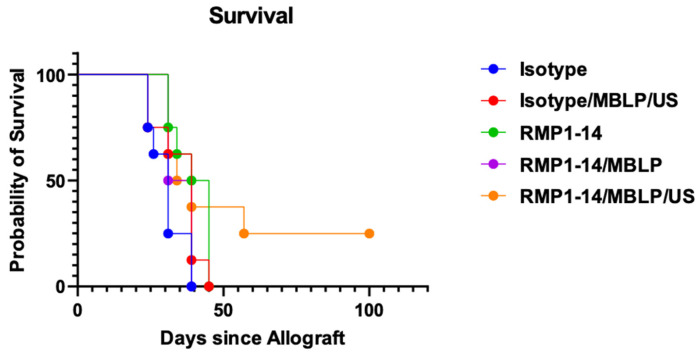
Kaplan-Meier survival analysis following tumor allograft. Overall survival probability plotted as a function of days since allograft for all treatment groups: Isotype, Isotype/MBLP/US, RMP1-14, RMP1-14/MBLP, RMP1-14/MBLP/US.

Using the AMI HT/HTX bioluminescence optical imager, no statistical difference in tumor size was observed between the control and treated groups (Figure 1a). Mouse weight also showed no significant differences between groups throughout treatment (Figure 1b). Detailed records were kept to document the reason for the euthanasia of each mouse. Mice treated with RMP1-14/MBLP/US showed the lowest ulceration rates, indicating reduced tumor growth and aggressiveness. Additionally, RMP1-14/MBLP/US was the only group to achieve complete tumor regression (Table 1). Although overall tumor size at endpoint remained similar across groups, RMP1-14/MBLP/US delayed tumor progression, with tumor burden remaining low for approximately 2–3 weeks post-treatment before regrowth, compared to only 3–7 days of growth delay following treatment with RMP1-14 alone. A Kaplan-Meier plot was used to show the probability of survival for each treatment, according to predefined criteria. Statistical significance was observed in both the RMP1-14 (*p* = 0.013) and RMP1-14/MBLP/US (*p* = 0.047) groups compared with the isotype control (Figure 2). Mean survival time was increased only in the RMP1-14/MBLP/US-treated group (Table 2a,b). The RMP1-14/MBLP/US group had the highest hazard ratio across all groups and was the only group to show a statistically significant HR compared to isotype control (HR 3.32, 95% CI 1.04–10.56: Table 2c), indicating a relative survival advantage of MBLP-mediated US delivery over isotype treatment.

### 3.2. MBLP/US Enhances RMP1-14-Induced Tumor Necrosis

**Figure 3:**
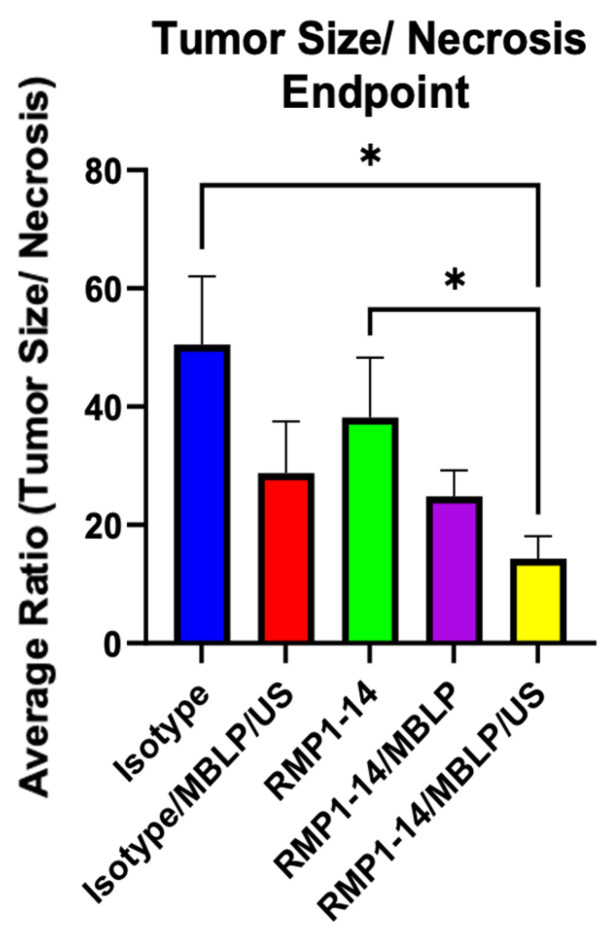
Tumor size-to-necrosis ratio at study endpoint. Bar graph depicting the average ratio of tumor size to necrosis area at endpoint across all treatment groups (Control, Isotype, Isotype/MBLP/US, RMP1-14, RMP1-14/MBLP, RMP1-14/MBLP/US). Lower ratios indicate greater relative necrosis. Data are presented as mean ± SEM. Significance: * *p* < 0.05.

Hematoxylin and eosin (H&E) staining was used to evaluate and quantify tumor necrosis for each treatment group (Figure 3). A lower tumor-to-necrotic area ratio indicates enhanced cell death. The RMP1-14/MBLP/US treatment group exhibited the highest degree of tumor necrosis compared to tumor size, which was significantly greater than that observed in the isotype and RMP1-14 alone treatment groups. Interestingly, tumors treated with US sonoporation had more dispersed necrosis consistent with increased extravascular delivery and evidence of US-induced vascular compromise. While RMP1-14/MBLP had a similar effect on survival to the isotype control, initially interpreted as reduced antibody delivery to the tumor, we observed that these tumors still showed necrosis comparable to RMP1-14 with and without MBLP-mediated US.

### 3.3. MBLP/US Increases RMP1-14 Intratumoral Bioavailability and Reduces Systemic Exposure

**Figure 4:**
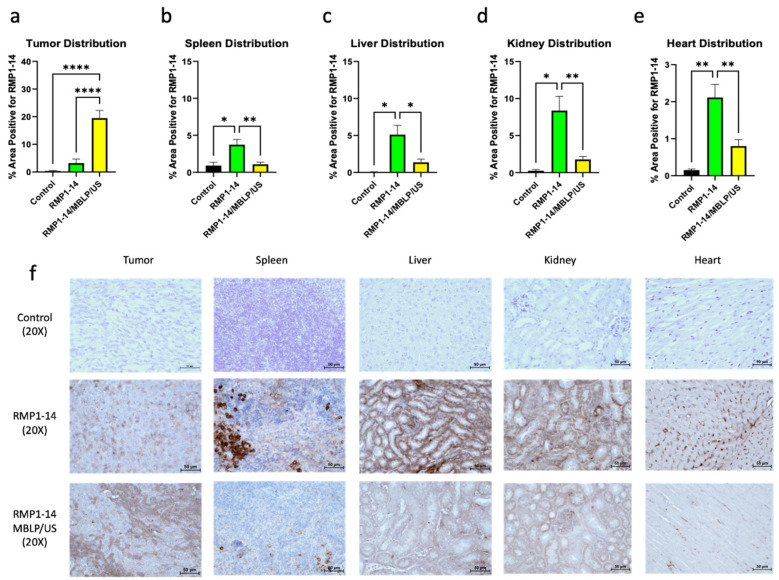
Tissue-specific distribution of RMP1-14 antibody assessed by immunohistochemistry. Bar graphs showing the percentage of area positive for rat IgG2a in (**a**) tumor, (**b**) spleen, (**c**) liver, (**d**) kidney, (**e**) heart, and (**f**) representative pictures of rat IgG2a immunohistochemical analysis of tumor, spleen, liver, kidney and heart for Saline Control, RMP1-14, and RMP1-14/MBLP/US groups. Significant increases in tumor accumulation and significant reductions in off-target organ distribution are observed with MBLP-mediated US delivery. Data are presented as mean ± SEM. Significance: * *p* < 0.05, ** *p* < 0.01, **** *p* < 0.0001.

Immunohistochemical staining of tumors and organs with an anti-rat IgG2a antibody demonstrated a significant increase in antibody intratumoral bioavailability in the group of mice receiving RMP1-14/MBLP/US (*p* < 0.0001) compared to RMP1-14 alone at 3 h (Figure 4a and Table 3). Systemic distribution of antibody in mice receiving RMP1-14/MBLP/US was notably reduced compared to RMP1-14 alone in the spleen (*p* = 0.0062), liver (*p* = 0.0200), kidneys (*p* = 0.0071), and heart (*p* = 0.0057) (Figure 4b–d and Table 3). Control mice that did not receive antibody showed no signal in the tumor or off-target organs, demonstrating the specificity of rat IgG2a for RMP1-14 detection and further validating this approach. This pronounced effect demonstrates that the novel microbubble/liposome (MBLP)-mediated US delivery selectively targets and concentrates therapeutics within the tumor microenvironment while minimizing off-target organ exposure.

### 3.4. MBLP/US Induces Transient Tumor Perfusion Enhancement

**Figure 5:**
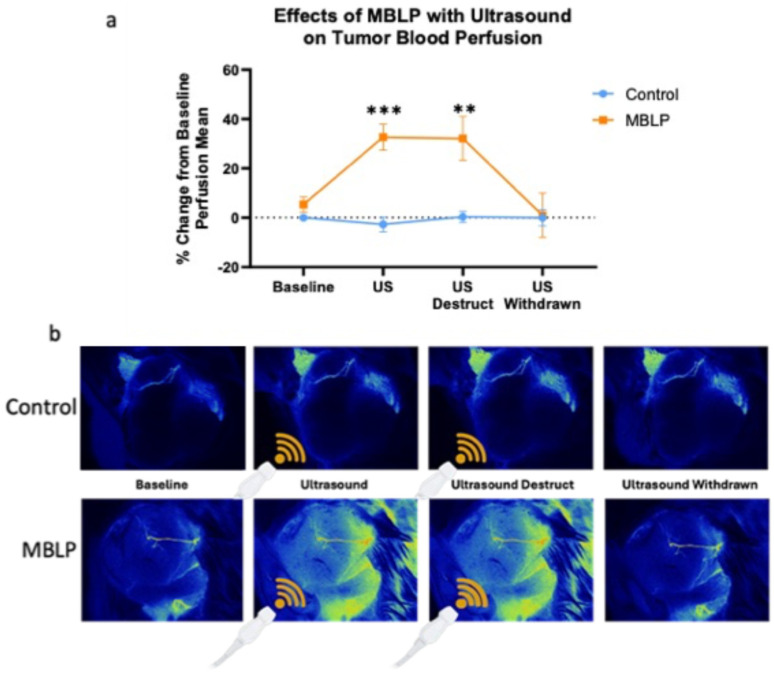
MBLP-enhanced ultrasound increases tumor blood perfusion. (**a**) Percent change from baseline tumor perfusion means across sequential ultrasound application stages (Baseline, Ultrasound, Ultrasound Destruction, Ultrasound Withdrawn) for Control and MBLP-treated groups (n mice/group = 8). MBLP administration, combined with ultrasound, resulted in a marked, transient increase in tumor perfusion compared with control. Data are presented as mean ± SEM. (**b**) Representative contrast-enhanced ultrasound images of tumor vasculature at each stage for Control (top row) and Microbubble (bottom row) groups, illustrating the spatiotemporal dynamics of perfusion enhancement. Significance: ** *p* < 0.01, *** *p* < 0.001.

To evaluate the effects of MBLP-mediation on tumor blood perfusion, Laser Speckle Imaging (LSI) was employed. Only a minimal increase in blood flow was observed following injection; however, neither MBLP alone nor US alone showed significant changes. In contrast, application of US in mice injected with MBLP increased tumor blood perfusion by 30% above baseline levels, relative to controls (*p* = 0.0001). No significant differences were detected between diagnostic-frequency US with or without destruction pulses, indicating that low frequency is adequate to increase intratumoral blood flow (Figure 5a,b). Enhanced perfusion across the entire tumor surface, even at the furthest distance from the US probe, suggests that the tumor core was sufficiently perfused. Although not statistically significant, tumor blood flow dropped just below baseline immediately after removal of the ultrasound probe, suggesting a rapid loss of hemodynamic effect unlikely due to increased temperature of the sonoporated tissue. Taken together, these findings indicate that MBLP-mediated ultrasound induces a substantial increase in tumor blood perfusion that cannot be achieved with ultrasound alone, consistent with increased antibody delivery and the dispersion of necrotic tissue.

## 4. Discussion

In this study, we demonstrate that microbubble/liposome-mediated ultrasound (MBLP/US) delivery of an anti-PD-1 antibody enhances therapeutic outcomes in an immunocompetent, syngeneic mouse model of colorectal cancer while simultaneously improving the systemic ICI safety profile compared with conventional intravenous monotherapy. Despite comparable early tumor burden across groups as assessed by bioluminescence imaging, both RMP1-14 and RMP1-14/MBLP/US significantly improved survival relative to isotype control (*p* = 0.013 and *p* = 0.047, respectively). RMP1-14/MBLP/US achieved a mean survival of 57.8 days, more than twice that of RMP1-14 alone (26.5 days) and more than threefold that of the isotype control (18.1 days). Although not seen as statistically significant using the Kaplan Meier method, it is interesting to note that RMP1-14/MBLP/US was the only group in which complete tumor regression was observed (2 out of 8 mice), exhibited the lowest ulceration prevalence among all treatment arms, and had the highest hazard ratio against standard therapy (HR 1.42; RMP1-14 alone vs. RMP1-14/MBLP/US), collectively indicating some clinical advantage conferred by US-mediated MBLP delivery. Of note, the RMP1-14/MBLP group had survival similar to the isotype and necrotic profiling like the RMP1-14 alone group, possibly indicating an insufficient dose of RMP1-14 delivered to the tumor. This indicates that the MBLP forms a protective shield around the cargo, which was shown by our lab to be beneficial in protecting viral load from immune inactivation. These findings align with and extend our laboratory’s prior reports that ultrasound-stimulated microbubbles can potentiate anti-PD-1 checkpoint responses, highlighting significantly improved intratumoral bioavailability via intravenous administration as a potentially beneficial strategy to enhance the efficacy of immune checkpoint inhibition in solid tumors.

Although we did not observe significant changes in tumor volume or in IHC assessment of infiltrative CD4+, CD8+, and CD25+ cells among the treatment groups at the terminal endpoint, histological assessment confirmed a substantial increase in intratumoral necrosis. In the context of ICI, the development of intratumoral necrosis has been associated with successful immune activation that induces tumor cell death and can be accompanied by pseudoprogression, in which tumor burden initially appears to increase before subsequently regressing [40,41]. H&E staining revealed that RMP1-14/MBLP/US produced the greatest degree of tumor necrosis at the experimental endpoint, significantly exceeding that observed in the isotype, isotype/MBLP/US, RMP1-14 alone, and RMP1-14/MBLP groups. Importantly, the RMP1-14/MBLP group, which received antibody-loaded MBLP without acoustic activation, showed necrosis levels comparable to RMP1-14 alone, confirming that microbubbles require ultrasound cavitation to achieve their full therapeutic potential. The pronounced necrosis in the RMP1-14/MBLP/US group indicates that superior antitumor effects are attributable to enhanced local RMP1-14 exposure within the TME, rather than to intrinsic differences in tumor growth kinetics or systemic toxicity. This is consistent with the well-established principle that physical modulation of tumor vasculature and interstitial transport can convert a partially effective immune checkpoint inhibitor into a more curative intervention, particularly in settings where baseline antibody penetration into the TME is a primary limiting factor.

Our IHC data directly support a delivery-based mechanism by demonstrating that MBLP-mediated ultrasound markedly reshapes the biodistribution of RMP1-14. Three hours after dosing, tumors from mice treated with RMP1-14/MBLP/US exhibited a pronounced increase in anti-rat IgG2a-positive area (19.5%) compared to those receiving RMP1-14 alone (3.2%; *p* ≤ 0.0001), confirming that acoustic cavitation substantially augments intratumoral antibody bioavailability. Critically, this local enrichment occurred in parallel with a significant reduction in antibody distribution in major clearance organs, such as the spleen (*p* = 0.0062), liver (*p* = 0.0200), kidneys (*p* = 0.0071), and heart (*p* = 0.0057). Tumors and organs collected from control mice receiving saline showed little to no DAB signal, validating the specificity of the IHC analysis. This spatial redistribution simultaneously concentrates antibody in the sonicated tumor while substantially reducing off-target organ deposition, thereby differing fundamentally from systemic dose-escalation strategies, which rely on a conventional therapeutic index paradigm. Prior reports of UTMD achieving focal macromolecular delivery modify only one side of the therapeutic index equation. However, our innovative microbubble/liposome co-formulation significantly reduces systemic exposure. Our observations demonstrate that utilizing MBLP/US co-formulation shifts the profile of RMP1-14 toward tumor accumulation rather than systemic organ exposure, representing a promising strategy to improve the therapeutic index of ICI, though formal demonstration will require paired toxicological and efficacy validation in future studies. Our data support a model in which MBLP-mediated ultrasound has the potential to improve the therapeutic and safety index of RMP1-14 by reshaping its biodistribution toward the TME without altering the antibody dose or molecular structure, consistent with reduced systemic exposure.

Laser speckle imaging provides mechanistic insight into the hemodynamic basis of this delivery enhancement. MBLP injection or ultrasound alone produced only minimal changes in tumor blood flow, whereas their combination increased tumor perfusion by approximately 30% above baseline (*p* = 0.0001), an effect absent in saline-injected controls. Along with this 30% increase in tumor blood flow, the LSI imaging indicated an increase in vascular perfusion, which could be due to increased vascular permeability caused by elevated capillary pressure and vessel distention, as well as an increased number of perfused vessels, or vascular density. Notably, no significant difference was observed between low-mechanical-index ultrasound with or without microbubble destruction pulses, suggesting that stable cavitation, rather than inertial cavitation alone, may be sufficient to generate the transient hemodynamic window exploited for drug delivery. Tumor blood flow returned rapidly to baseline levels immediately after cessation of sonication, arguing against a sustained thermal mechanism and instead implicating short-lived biomechanical effects, most plausibly cavitation-mediated increases in microvascular permeability, transient vasodilation, or alterations in red blood cell flux, as the principal drivers of enhanced transport. Interpreted alongside our IHC and necrosis data, this transient perfusion surge likely facilitates greater extravasation and deeper penetration of circulating RMP1-14 into the TME, which in turn translates into more extensive tumor necrosis.

The present findings integrate and extend prior work on ultrasound-augmented immunotherapy by directly linking survival, histological necrosis, antibody biodistribution, and tumor perfusion within a single experimental framework. Prior studies have established that UTMD can modulate the TME, enhance delivery of chemotherapeutics or antibodies, and augment systemic antitumor immunity, in part through acoustic cavitation-mediated promotion of dendritic cell maturation, CD8+ T cell infiltration, and macrophage polarization toward an anti-tumor phenotype [27,42,43]. However, to our knowledge, few studies have concurrently quantified changes in intratumoral antibody concentrations and off-target organ exposure alongside functional perfusion imaging in an immunocompetent host [44,45,46]. By demonstrating that RMP1-14/MBLP/US increases intratumoral antibody bioavailability by approximately six-fold, reduces accumulation in major clearance organs by up to 94%, and induces a reversible perfusion surge that is contingent on the combination of MBLP and US, our data provide mechanistic support for the use of MBLP-mediated ultrasound as a platform to selectively widen the therapeutic window of PD-1 checkpoint inhibition without having to alter the chemistry, manufacturing, or commercial distribution of the antibodies whatsoever.

This study has several limitations that identify important directions for future investigation. First, antibody biodistribution was assessed at a single early time point (3 h post-single dosing); additional longitudinal sampling would be required to fully characterize the kinetics of tumor and organ exposure and to confirm that the intratumoral advantage conferred by MBLP/US is maintained across the dosing interval. Second, although our perfusion data support a hemodynamic mechanism, higher-resolution vascular imaging and direct measurements of interstitial pressure or hydraulic conductance would be needed to distinguish among candidate biophysical processes, including sonoporation-mediated endothelial pore formation, transient vasodilation, and alterations in intravascular blood velocity. Our LSI and IHC data prompt future studies to assess vascular permeabilization, including the Evans blue dye extravasation assay, to further validate that MBs enhance ultrasound-mediated modulation of vascular infrastructure. Future analyses of tumor temperature could rule out the possible effects of heat induction on tumor blood flow; results would still be limited to extratumoral extrapolation. Third, a full therapeutic dose of RMP1-14 was employed across all treatment groups; we hypothesize that the survival benefit of RMP1-14/MBLP/US would be amplified under subtherapeutic dosing conditions, thereby more clearly isolating the contribution of enhanced delivery. Future studies should address this by reducing the anti-PD-1 dose across all groups to negate oversaturation that may have dampened group differences and more clearly highlight the increased intratumoral concentration observed in the RMP1-14/MBLP/US group. Antibody integrity following MBLP preparation by manufacturers’ instructions was not assessed, nor was the percentage of free vs. encapsulated antibody. The discrepancy between necrosis and survival in the RMP1-14/MBLP group may reflect liposomal sequestration of antibody without US-triggered release, limiting free antibody availability for sustained PD-1 receptor engagement and durable immune activation, a mechanistic question we plan to address in future studies. Finally, while stable mouse weight and reduced off-target organ staining are consistent with a favorable systemic safety profile, formal toxicology studies, including immune profiling for irAEs, will be essential prior to clinical translation to further verify that the data are not simply time-dependent.

Despite these limitations, our results support the concept that MBLP-mediated ultrasound can specifically enhance intratumoral delivery of checkpoint immune inhibitors, leading to greater tumor necrosis and improved survival without proportionally increasing systemic organ exposure. This approach could also be considered translatable to improve the distribution of antibody-based therapeutics, such as antibody-drug conjugates (ADCs) or Bi-Specific T Cell Engagers (BiTEs). Future studies will be required to optimize treatment parameters for clinical-scale tumors and to evaluate delivery efficiency in deeper and larger lesions. Rather than escalating systemic doses associated with increased adverse effects, MBLP/US offers a potential approach to improve intratumoral delivery in solid tumors characterized by poor perfusion, high interstitial pressure, or limited immune infiltration. Future studies integrating longitudinal pharmacokinetic imaging, immune cell profiling, and combination regimens will further clarify the optimal parameters and patient populations for MBLP-mediated ultrasound as an adjunct to current immunotherapy.

## Figures and Tables

**Table 1:** Summary of tumor outcome measures by treatment group. Tumor outcomes are reported for each treatment group, including the percentage of tumors that became ulcerated, the percentage that reached the protocol-defined size constraint requiring removal (2000 mm^3^), and the number of tumors that underwent complete regression. Only the RMP1-14/MBLP/US combination group produced full tumor regressions (n = 2).

Groups	% Ulcerated Tumors	% Tumors Reaching Protocol Size Constraint	Number of Tumors Regressed
Isotype	50%	50%	0
Isotype/MBLP/US	25%	75%	0
RMP1-14	40%	60%	0
RMP1-14/MBLP	25%	75%	0
RMP1-14/MBLP/US	25%	50%	2 (full regression)

**Table 2:** (**a**) Pairwise log-rank *p*-values from Kaplan-Meier survival comparisons. Statistical comparisons of survival distributions between all treatment group pairs. Significance threshold: * *p* < 0.05. Significant differences were observed between RMP1-14 vs. Isotype (*p* = 0.013) and RMP1-14/MBLP/US vs. Isotype (*p* = 0.047). (**b**) Average survival time by treatment group. Mean survival time (days) ± standard deviation for each treatment group. The RMP1-14/MBLP/US group demonstrated the longest mean survival (57.75 ± 19.39 days), compared to the isotype control (18.13 ± 1.71 days). (**c**) Pairwise hazard ratios from survival analysis. Cox proportional hazard ratios (HR) with standard error of the mean (SEM) and 95% confidence intervals (lower and upper confidence limits, LCL/UCL) for all pairwise group comparisons. HR > 1 indicates greater hazard (shorter survival) in the numerator group relative to the group in the denominator. Significance threshold: * *p* < 0.05.

(**a**)				
**KM Survival Comparisons**	***p*** **-Value**			
All	0.062			
RMP1-14 vs. RMP1-14/MBLP/US	0.441			
RMP1-14 vs. RMP1-14/MBLP	0.145			
RMP1-14 vs. Isotype/MBLP/US	0.475			
RMP1-14 vs. Isotype	0.013 *			
RMP1-14/MBLP/US vs. Isotype	0.047 *			
RMP1-14/MBLP/US vs. Isotype/MBLP/US	0.299			
RMP1-14/MBLP/US vs. RMP1-14/MBLP	0.346			
Isotype vs. RMP1-14/MBLP	0.309			
Isotype vs. Isotype/MBLP/US	0.126			
Isotype/MBLP/US vs. RMP1-14/MBLP	0.606			
(**b**)				
**Groups**	**Average Survival Time**			
All	30.08			
Dead/Alive = Isotype	18.13			
Dead/Alive = Isotype/MBLP/US	22.50			
Dead/Alive = RMP1-14	26.50			
Dead/Alive = RMP1-14/MB	21.00			
Dead/Alive = RMP1-14/MBLP/US	57.75			
(**c**)				
**Hazard Ratios (Dead/Alive)**	**HR**	**SEM**	**LCL (95%)**	**UCL (95%)**
“Isotype”/“Isotype/MBLP/US”	1.86	0.62	0.55	6.26
“Isotype”/“RMP1-14”	2.34	0.60	0.73	7.52
“Isotype”/“RMP1-14/MBLP”	1.55	0.75	0.35	6.81
“Isotype”/“RMP1-14/MBLP/US”	3.32 *	0.59	1.04	10.56
“Isotype/MBLP/US”/“RMP1-14”	1.25	0.49	0.48	3.28
“Isotype/MBLP/US”/“RMP1-14/MBLP”	0.83	0.67	0.22	3.12
“Isotype/MBLP/US”/“RMP1-14/MBLP/US”	1.78	0.48	0.69	4.59
“RMP1-14”/“RMP1-14/MBLP”	0.66	0.65	0.18	2.39
“RMP1-14”/“RMP1-14/MBLP/US”	1.42	0.45	0.58	3.46
“RMP1-14/MBLP”/“RMP1-14/MBLP/US”	2.14	0.65	0.60	7.62

**Table 3:** Tissue distribution of RMP1-14 antibody with and without microbubble-enhanced ultrasound delivery. Percentage of tissue area positive for rat IgG2a (RMP1-14) in tumor, spleen, liver, kidney, and heart for the RMP1-14 and RMP1-14/MBLP/US treatment groups. MBLP/US delivery significantly increased tumor accumulation (3.20% vs. 19.50%, *p* = 0.0003) while substantially reducing off-target distribution to the spleen, liver, kidney, and heart. Significance: * *p* < 0.05, ** *p* < 0.01, **** *p* < 0.0001.

Tissue Distribution	RMP1-14	RMP1-14/MBLP/US	*p* Value
Tumor	3.20%	19.47%	<0.0001 ****
Spleen	5.84%	0.13%	0.0062 **
Liver	7.24%	0.35%	0.0200 *
Kidney	11.46%	1.74%	0.0071 **
Heart	1.10%	0.36%	0.0057 **

## Data Availability

Data presented in this study is contained within the article. Further inquiries can be directed to the corresponding author.

## Abbreviations

Colorectal cancer (CRC), immune checkpoint inhibitors (ICIs), immune-related adverse events (irAEs), intravenous (IV), microbubbles (MB), microbubble/liposomes (MBLP), microsatellite stable (MSS), mismatch repair-deficient (dMMR), non-small cell lung cancer (NSCLC), tumor microenvironment (TME), ultrasound (US), ultrasound-targeted microbubble delivery (UTMD).
